# Budget impact analysis of durvalumab consolidation therapy vs no consolidation therapy after chemoradiotherapy in stage III non–small cell lung cancer in the context of the Chilean health care system

**DOI:** 10.1371/journal.pone.0307473

**Published:** 2024-07-26

**Authors:** Nicolás Armijo, Constanza Salas, Nazareth Espinoza, Manuel Espinoza, Carlos Balmaceda

**Affiliations:** 1 Epsilon Research, Santiago, Chile; 2 Departamento de Salud Pública ‐ Pontificia Universidad Católica de Chile, Santiago, Chile; Inha University Hospital, REPUBLIC OF KOREA

## Abstract

**Background:**

Durvalumab, used as consolidation immunotherapy, has shown to improve survival in patients with stage III non-small cell lung cancer who respond to chemoradiotherapy, based on the most recent follow-up of PACIFIC. The Chilean healthcare system provides access to certain immunotherapies for this condition. The present study sought to estimate the budget impact of durvalumab versus standard of care in the context of the Chilean healthcare system.

**Research design and methods:**

A partitioned survival model was adapted to compare two strategies: durvalumab as consolidation therapy and standard of care for treating stage III NSCLC. The number of patients eligible for treatment was estimated using published incidence data and modeled for a 5-year time horizon. Model inputs were based on published literature, and the duration of treatment was estimated using survival curves obtained from PACIFIC. Costs were estimated in Chilean pesos (CLP) and converted to USD dollars using an exchange rate of USD 1  =  CLP 827. Scenario analyses were performed to assess different subsequent therapy splits, variations in the target population and dosage of durvalumab.

**Results:**

Durvalumab uptake projected total costs ranging from USD 1.27 in Year 1 to 8.5 million in Year 5 from the public perspective. From the private perspective, the budget impact for the first year is USD 1.3 million to USD 3 million for 2028. This difference relies mostly on the lower number of patients treated. Both perspectives anticipated cost savings over the time horizon through reduced monitoring, adverse events, and end-of-life expenses.

**Conclusions:**

This study demonstrates that the inclusion of Durvalumab for NSCLC in Chile represents an investment in the Chilean health system. The incremental costs align with clinical benefits and potential savings in healthcare resource utilization. However, a comprehensive cost-effectiveness analysis is needed to evaluate its economic value thoroughly.

## Introduction

Lung cancer has been the leading cause of cancer incidence and mortality worldwide [1]. Non-small cell lung cancer (NSCLC) is the most frequent type, accounting for 80–85% of all lung cancers [2]. Historically, patients diagnosed with NSCLC in later stages have been treated with standard of care, consisting of surgical resection and (neo)- adjuvant chemotherapy or definitive chemoradiotherapy [3]. More recently, advances in immunotherapy have led to promising developments in treating NSCLC [3].

The treatment pathway depends on the characteristics of the cancer. For non-resectable and potentially resectable NSCLC in stage III, double platinum chemotherapy is indicated with radiotherapy. In contrast, resectable cancer is a candidate for surgery or a radiotherapy and chemotherapy scheme [3]. However, patients who have received chemoradiotherapy often show limited response in terms of median progression-free survival [4, 5]. In those cases, immunotherapy can enhance treatment outcomes if given early (as neoadjuvant treatment), synergistically (adjuvant setting), or as consolidation [6].

Chile has significantly improved access to cancer treatments in the last few years. Specifically, lung cancer was incorporated into the national health benefit plan GES (*garantías explícitas de salud)*. GES provides healthcare services for 87 health problems established by law [7], guaranteeing quality of care, timeliness and financial protection to all public and private insurer beneficiaries. Currently, the therapies included in GES cover diagnostic, monitoring, and treatment with chemoradiotherapy and chemotherapy. For NSCLC stage III, the treatment recommended is chemoradiotherapy with platinum (radiotherapy, etoposide, cisplatin, vinorelbine, gemcitabine and paclitaxel). The standard of care (SoC) for non-resectable and locally advanced is patient monitoring until progression [8].

Furthermore, immunotherapy is covered through a different reimbursement scheme called high-cost oncological drugs (DAC, *Drogas Oncológicas de Alto Costo in Spanish*). The DAC arsenal includes high-cost immunotherapies such as Pembrolizumab, Nivolumab and Atelozilumab [9].

Durvalumab is a selective PD-L1 inhibitor, first approved in 2018 on the efficacy demonstrated in the PACIFIC phase 3 trial (ClinicalTrials.gov identifier NCT02125461 [10]. It became the first PD-L1 inhibitor available for adjuvant treatment of patients with unresectable stage III NSCLC whose cancer has not progressed after definitive chemoradiotherapy. Durvalumab has demonstrated to improve median progression-free survival (16.8 months, [95% CI 13.0 to 18.1] versus placebo 5.6 months, HR 0.52, [95% CI 0.42 to 0.65]). Furthermore, the median time to death or distant metastasis was longer with durvalumab than with placebo (23.2 months vs. 14.6 months; P<0.001).

As immunotherapy continues to expand its indications, projecting the potential budgetary impact has become fundamental to inform resource allocation decisions. This study aimed to undertake a budget impact analysis of durvalumab as consolidation therapy after definitive chemoradiotherapy for patients with unresectable stage III NSCLC compared to the current standard of care from the Chilean public and private healthcare subsystems.

## Methods

We implemented a budget impact model following the principles of good practice elaborated by ISPOR [11]. We estimated the annual impact for a 5-yeartime horizon period. The target population of the study was adults diagnosed with stage III, unresectable, and locally advanced NSCLC, who have undergone chemoradiotherapy and who have not progressed in their disease. The population was estimated for the public and private segments of the Chilean health system.

### Population

Country-level estimates of population older than 15 years old were obtained from the projections calculated by the National Statistical Institute (*Instituto Nacional de Estadísticas*, INE). According to the registry of the National Health Fund (FONASA), the Chilean public national insurer, we estimated that 78.8% of the Chilean population, i.e., 12,785,819 people, are beneficiaries of the public sector [12]. This percentage is considered constant along the time horizon. On the other hand, we estimated that 2,581,845 people are privately insured, corresponding to 15.9% of the population, which is based on the information published by the Superintendence of Health [13].

Incidence of NSCLC over 15 years was estimated at 0.0206% according to GLOBOCAN’s last report [14]. Additionally, 16.3% were categorized in stage III, given local publication [15]. There is diverse evidence regarding the percentage of patients treated with chemoradiotherapy, with figures ranging from 54% to 81.4%. Therefore, given the uncertainty associated with this parameter, a base case estimate of 81.4% was employed based on sources where both chemoradiotherapy and surgery were available [16, 17]. However, recognizing the uncertainty surrounding this parameter, a scenario analysis was conducted using a value of 54%, drawing from sources where all patients were exclusively treated with chemoradiotherapy [18, 19]. Regardless of the source of information selected, only the subset that does not progress is eligible for durvalumab, which represents 67.7% [20].

Patients on treatment, patients free of progression, patients alive, and patients in TFST (time to first subsequent therapy) were modelled using a survival model according to the most recent 12-month follow-up data of the PACIFIC trial [21].

### Costs

Costs included drug acquisition, monitoring, administration, adverse events, and end-of-life care. They were measured in Chilean pesos (CLP) and converted into USD dollars using an exchange rate of USD 1  =  CLP 817 (mean of reported dollar rate between 1^st^ of January and 15^th^ of September 2023) [22]. Main sources of resource used and unit costs were verification cost study (*Estudio de verificación de costos*, EVC), Institutional Care Modality (*Modalidad atención institucional*, MAI) 2023 [23], CENABAST drug purchases [24], Public Market [25] and available list prices from pharmaceutical laboratories. A micro-costing approach was performed based on local and international literature to estimate the expected cost. The baskets included healthcare services for diagnosis, treatment, follow-up, and rehabilitation with a defined set of consultants, laboratory tests, images, surgeries, and drug therapy, among others [26].

In our model, we have made certain assumptions. We anticipate that the market share for durvalumab in the public sector would increase every year, starting with 15% in the first year and then 30%, 50%, 65%, and up to 70% in the fifth year. This assumption takes into account the potential access barriers that the Chilean public system may face when including Durvalumab coverage under the DAC scheme [27]. For the private sector, we assumed the uptake of durvalumab will reach 70% in the first year, increasing by 10% yearly, which implies 100% in years 4 and 5 (Table A in S1 Appendix). This assumption aligns with the expectation that the private payer will encounter lower access barriers from the financing and purchasing mechanism than the public sector [28–30].

A list of all model inputs is presented in Table 1.

**Table 1:** Parameters.

Epidemiology		
Parameter	Estimator	Source
NSCLC incidence (over 15 years)	0.0206%	GLOBOCAN 2020
% Proportion of stage III NSCLC patients	16.3%	Gonzalez et al
% Proportion of chemoradiotherapy patients	81.4%	Own estimation
	54%	Own estimation
% Proportion of non-progressors patients	67.6%	Eichkorn et al
**Proportion of patients who receive subsequent treatment**		
	Durvalumab	SoC
Pembrolizumab	4.00%	4.00%
Pemetrexed + Cisplatin	40.00%	40.00%
Pemetrexed + Carboplatin	40.00%	40.00%
Alectinib	4.00%	4.00%
Nivolumab	4.00%	4.00%
Atezolizumab	4.00%	4.00%
**Incidence of adverse events**	Durvalumab	SoC
Pneumonia	0.065	0.076
Anemia	0.033	0.060
Pneumonitis	0.033	0.015
Endocrinopathy	0.005	0.000
Hypokalemia	0.022	0.076
Hemoptysis	0.000	0.030
Radiation pneumonia	0.027	0.045
**Cost per treatment (unitary vial)**		
	FONASA	ISAPRE
Durvalumab (vial 500mg)	$ 2,426	$ 2,788
Durvalumab (vial 120mg)	$ 582	$ 669
Total IV administration	$ 20	$ 124
**Cost per adverse event (each event)**		
Pneumonia	$ 823	$ 2.537
Anemia	$ 482	$ 1.533
Pneumonitis	$ 2.294	$ 6.145
Endocrinopathy	$ 120	$ 526
Hypokalemia	$ 46	$ 139
Hemoptysis	$ 2,294	$ 6,145
Radiation pneumonia	$ 2,294	$ 6,145
Monitoring cost PFS		
Total year 1	$ 423	$ 2,081
Total year 2	$ 423	$ 2,081
Total year 3–5	$ 423	$ 2,081
Monitoring cost PPS		
Total year 1	$ 971	$ 5,685
Total year 2	$ 971	$ 5,685
Total year 3–5	$ 971	$ 5,685
Terminal costs (end of life care), monthly		
End of life care (advanced cancer)	$ 138	$ 398
Care for pain relief within cancer progression	$ 57	$ 123
Total cost (monthly)	$ 195	$ 520
**Cost of subsequent treatment per patient (FONASA)**		
Pembrolizumab	$ 77,935	$ 77,935
Pemetrexed + Cisplatin	$ 815	$ 815
Pemetrexed + Carboplatin	$ 972	$ 972
Alectinib	$ 280	$ 280
Nivolumab	$ 50,597	$ 50,597
Atezolizumab	$ 33,967	$ 33,967
Total cost subsequent treatment	$ 7,226	$ 7,226
**Cost of subsequent treatment per patient (ISAPRE)**		
Pembrolizumab	$ 155,994	$ 155,994
Pemetrexed + Cisplatin	$ 3,399	$ 3,399
Pemetrexed + Carboplatin	$ 3,751	$ 3,751
Alectinib	$ 464	$ 464
Nivolumab	$ 74,996	$ 74,996
Atezolizumab	$ 49,541	$ 49,541
Total cost subsequent treatment	$ 14,100	$ 14,100

All costs are expressed in USD.

### Scenario analysis

The base case considers that 80% of patients will receive chemotherapy as a subsequent therapy, and 20% will be administrated with immunotherapy. Additionally, this base case assumes the proportion of patients candidates for chemoradiotherapy is 81.4%. The first scenario analysis assumes that only 60% will receive chemotherapy as a subsequent treatment, and the rest will be covered by immunotherapy (Table B in S1 Appendix). Conversely, the second scenario considers 54% of the population as candidates for chemoradiotherapy and 80/20 chemotherapy and immunotherapy, respectively, as subsequent therapy. Lastly, the third scenario used the same assumptions as the second scenario assuming a fixed dosage for durvalumab (1500 mg every 4 weeks).

### Ethics statement

The evaluation does not involve human participants, as it is exclusively based on data derived from published literature and anonymized data already available in the public domain. The numbers of individuals mentioned correspond to a simulated cohort. Consequently, ethical approval was not deemed necessary, given the absence of direct involvement of individuals in the study, and all data used were publicly available and anonymized. All relevant data are within the paper and its Supporting Information files.

## Results

### Base case for public perspective

The target population considering NSCLC patients stage III non-resectable and locally advanced for the public sector results in 238 patients for the first year, from which 38 will be eligible for durvalumab (Table 2). The incorporation of durvalumab decreases subsequent costs by $17,535 in the first year, reaching savings of $90,593 by 2028. Durvalumab also incurs savings for adverse events, monitoring, and end-of-life costs. Acquisition and administration costs for durvalumab are the main cost drivers.

**Table 2:** Base case results in public perspective.

	2024	2025	2026	2027	2028
Total number of patients who have been treated by the end of each year	238	240	242	244	247
Number of patients who receive durvalumab	36	72	121	159	172
**SoC**					
Drug acquisition cost, USD	$ -	$ -	$ -	$ -	$ -
Administration cost, USD	$ -	$ -	$ -	$ -	$ -
Adverse event cost, USD	25,084	38,221	38,559	38,885	39,195
Monitoring cost, USD	821,360	2,371,463	3,608,449	4,578,655	5,367,614
End of life cost, USD	20,585	145,095	379,280	688,512	1,048,177
Subsequent treatment cost, USD	293,221	667,635	559,773	437,414	350,624
Total cost	$1,160,250	$3,222,413	$4,586,061	$5,743,467	$6,805,610
**Durvalumab**					
Drug acquisition cost, USD	1,305,405	3,390,744	5,955,500	8,371,649	9,664,604
Administration cost, USD	7,213	18,736	32,909	46,260	53,404
Adverse event cost, USD	24,174	36,101	34,900	33,876	33,542
Monitoring cost, USD	810,425	2,331,131	3,531,523	4,469,661	5,252,533
End of life cost, USD	4,522	14,193	21,121	25,203	27,806
Subsequent treatment cost, USD	275,685	617,826	480,263	335,926	260,031
Total cost	$2,427,425	$6,408,730	$10,056,215	$13,282,575	$15,291,920
**Incremental difference**					
Drug acquisition cost, USD	1,305,405	3,390,744	5,955,500	8,371,649	9,664,604
Administration cost, USD	7,213	18,736	32,909	46,260	53,404
Adverse event cost, USD	-910	-2,120	-3,660	-5,009	-5,653
Monitoring cost, USD	-10,935	-40,331	-76,926	-108,994	-115,081
End of life cost, USD	-16,063	-130,902	-358,159	-663,309	-1,020,371
Subsequent treatment cost, USD	-17,535	-49,809	-79,511	-101,488	-90,593
**Budget Impact**	$1,267,175	$3,186,317	$5,470,153	$7,539,109	$8,486,310

### Scenario analysis

Results of scenario analysis are presented in Table 3. The change in the proportions of subsequent treatment to 60% chemotherapy and 40% immunotherapy resulted in a minor difference (1%). The second scenario, where the target population is assumed to include only 54% of patients treated with chemotherapy, resulted in a budget impact decrease of 34% versus the base case along the time horizon. The last scenario considers the target population calculated in the previous case and assumes that 80% of patients will receive chemotherapy as subsequent therapy, and the rest will be administered immunotherapy. The budget impact is reduced by 33.5% compared to the base case. This results in $425,079 savings in the first year and $2,840,253 for the fifth year.

**Table 3:** Scenario analysis (incremental costs) in public perspective.

	2024	2025	2026	2027	2028
**Scenario 1**					
Total number of patients who have been treated by the end of each year	238	240	242	244	247
Drug acquisition cost, USD	1,305,405	3,390,744	5,955,500	8,371,649	9,664,604
Administration cost, USD	7,213	18,736	32,909	46,260	53,404
Adverse event cost, USD	-910	-2,120	-3,660	-5,009	-5,653
Monitoring cost, USD	-10,935	-40,331	-76,926	-108,994	-115,081
End of life cost, USD	-16,063	-130,902	-358,159	-663,309	-1,020,371
Subsequent treatment cost, USD	-31,690	-92,479	-148,091	-190,007	-171,257
Total cost	1,253,020	3,143,647	5,401,573	7,450,589	8,405,646
**Scenario 2**					
Total number of patients who have been treated by the end of each year	156	157	160	160	162
Drug acquisition cost, USD	857,999	2,228,622	3,914,351	5,502,405	6,352,221
Administration cost, USD	4,741	12,315	21,630	30,405	35,101
Adverse event cost, USD	-598	-1,393	-2,405	-3,292	-3,716
Monitoring cost, USD	-7,187	-26,508	-50,561	-71,638	-75,639
End of life cost, USD	-10,558	-86,038	-235,406	-435,971	-670,656
Subsequent treatment cost, USD	-11,525	-32,738	-52,260	-66,705	-59,544
Total cost	$832,872	$2,094,260	$3,595,349	$4,955,204	$5,577,768
**Scenario 3**					
Total number of patients who have been treated by the end of each year	156	157	160	160	162
Drug acquisition cost, USD	869,594	2,258,739	3,967,247	5,576,761	6,438,061
Administration cost, USD	2,371	6,157	10,815	15,202	17,550
Adverse event cost, USD	-598	-1,393	-2,405	-3,292	-3,716
Monitoring cost, USD	-7,187	-26,508	-50,561	-71,638	-75,639
End of life cost, USD	-10,558	-86,038	-235,406	-435,971	-670,656
Subsequent treatment cost, USD	-11,525	-32,738	-52,260	-66,705	-59,544
**Budget Impact**	$842,096	$2,118,219	$3,637,430	$5,014,357	$5,646,057

### Base case private perspective

The target population considering NSCLC patients stage III non-resectable and locally advanced for the private sector result in 48 patients for the first year, from which 34 will be eligible for durvalumab (Table 4). Following the same split for subsequent therapies than in the public perspective, durvalumab incurs savings versus SoC in this category, next to adverse events, monitoring, and end-of-life costs. The budget impact for 2024 is USD 1,349,002. Scenario analysis for the private perspective is reported in Table C in (S1 Appendix).

**Table 4:** Base case results in private perspective.

	2024	2025	2026	2027	2028
Total number of patients who have been treated by the end of each year	48	49	49	49	50
Number of patients who receive durvalumab	34	39	44	49	50
**SoC**					
Drug acquisition cost, USD	$ -	$ -	$ -	$ -	$ -
Administration cost, USD	$ -	$ -	$ -	$ -	$ -
Adverse event cost, USD	14,242	21,700	21,892	22,077	22,253
Monitoring cost, USD	887,706	2,631,990	4,036,162	5,136,846	6,029,988
End of life cost, USD	2,557	8,093	12,309	15,082	16,980
Subsequent treatment cost, USD	121,228	262,306	219,464	171,551	137,611
Total cost	$1,025,733	$2,924,088	$4,289,827	$5,345,556	$6,206,832
**Durvalumab**					
Drug acquisition cost, USD	1,413,951	2,449,725	2,795,132	3,145,134	3,290,343
Administration cost, USD	42,495	73,624	84,005	94,524	98,888
Adverse event cost, USD	11,870	18,235	17,947	17,645	17,679
Monitoring cost, USD	816,789	2,419,204	3,744,907	4,818,894	4,818,894
End of life cost, USD	2,004	6,522	9,982	12,179	13,678
Subsequent treatment cost, USD	87,626	198,771	163,559	133,788	121,586
Total cost	$2,374,735	$5,166,081	$6,815,533	$8,222,165	$9,274,141
**Incremental difference**					
Drug acquisition cost, USD	1,413,951	2,449,725	2,795,132	3,145,134	3,290,343
Administration cost, USD	42,495	73,624	84,005	94,524	98,888
Adverse event cost, USD	-2,372	-3,465	-3,945	-4,432	-4,574
Monitoring cost, USD	-70,917	-212,785	-291,255	-317,952	-298,022
End of life cost, USD	-553	-1,572	-2,327	-2,902	-3,302
Subsequent treatment cost, USD	-33,601	-63,535	-55,905	-37,763	-16,025
**Budget Impact**	$1,349,002	$2,241,993	$2,525,706	$2,876,609	$3,067,308

## Discussion

This study aimed to estimate the budget impact analysis of incorporating durvalumab for the treatment of patients diagnosed with stage III NSCLC, non-resectable and locally advanced compared to the currently available practice in Chile, from the perspective of the public and private healthcare system. The main drivers of costs for durvalumab are acquisition and administration costs for both perspectives along the time horizon. The budget impact analysis reaches USD 1,267,175 in the first year to USD 8,486,310 for the fifth year for the public system and USD 1,349,002 (year 1) and USD 3,067,308 (year 5) for the private sector. The difference in budget impact between both systems relies on the number of patients accessing the therapy. Adding durvalumab generates savings and can improve patient outcomes, which can be attributed to decreased monitoring, adverse events, and end-of-life costs.

This analysis shows that the implementation of durvalumab would be reasonable when considered within the healthcare expenditures of other high-cost drugs. Regarding the resources FONASA executed in 2022, a total of USD 10,656,865 were allocated for PD-L1 inhibitors through the DAC. This means the budget impact of durvalumab would represent 11.9% of this expenditure. When comparing the introduction of durvalumab to the total budget of DAC for 2022, this represents 3.3% of the budget (Total budget for 2022 was USD 38,978,657) [27].

Our study also presents some limitations. First, compliance was assumed to be 100% for all treatments, which may not reflect real-world practice. Second, given the limited literature available for the region and the heterogeneity of data sources regarding subsequent treatment when administering durvalumab in the Chilean context, we faced important uncertainty. Nevertheless, we explored different scenarios to mitigate this drawback. The first scenario analysis assessed the expected cost variations and budget impact through different assumptions on the frequency of subsequent treatments. In addition, two additional analyses were performed. The second scenario was designed to examine the impact of using different sources of information on the calculation of the target population, particularly on the proportion of patients who had already received chemoradiotherapy. Lastly, the third scenario explored using a fixed dose application instead of weight-based dosing for durvalumab as done for the base case.

This work represents the first budget impact analysis undertaken in the Latin America region. Previous studies have been published assessing the cost-effectiveness of durvalumab in the US [31–33], China [34], the UK [35], Italy [36, 37], and Switzerland [38]. Despite differences in intervention costs, willingness-to-pay thresholds, and insurance policies, durvalumab was found to be cost-effective compared to no consolidation therapy. It should be noted that these findings cannot be generalized to other jurisdictions.

Although one study conducted a budget impact analysis showing a 0.8% increase in direct medical costs associated with all cancers, projected cost savings over time can help mitigate this budgetary impact [31]. This outcome aligns with our findings, where we estimated cost savings in terms of adverse events, monitoring, end-of-life care, and subsequent treatment.

Among the strengths of this study is that the analysis undertakes a conservative scenario regarding subsequent therapy. Further savings could be obtained by exploring shorter chemotherapy cycles resembling the protocol used in the PACIFIC trial [39]. Furthermore, long-term benefits are not reflected in this study, thus undertaking a cost-effectiveness analysis to better capture the value of this technology. This could also provide the opportunity to explore population subgroups. As results suggest in the pivotal trial, patients presenting PD-L1 show favourable PFS (HR, 0.80; 95% CI, 0.53 to 1.20) [21]. Therefore, patients expressing PD-L1 might incur further benefits under the therapy of durvalumab.

## Conclusion

The introduction of durvalumab as a therapy for stage III non-resectable and locally advanced NSCLC represents an increase in the overall spending on cancer treatment in Chile. However, the budget impact is expected to result in cost savings in monitoring, adverse events, and end-of-life costs in public and private subsystems. These savings represent a decrease in demand for certain healthcare services, which provide further benefits than the monetary value assessed in this study. Further cost-effectiveness analysis is warranted to assess the efficiency associated with durvalumab within the Chilean healthcare system.

## Supporting information

S1 Appendix (ZIP)

S1 Data (XLSX)
