# Supplementary material for: Budget impact analysis of durvalumab consolidation therapy vs no consolidation therapy after chemoradiotherapy in stage III non–small cell lung cancer in the context of the Chilean health care system
Source: PLoS One. 2024 Jul 26;19(7):e0307473. doi: 10.1371/journal.pone.0307473 (PMC11280244; doi:10.1371/journal.pone.0307473)
Supplement: S1 Appendix — (ZIP) [file pone.0307473.s001.zip › S1 Table A.docx]

**S1 Appendix. Table A. Market Shares**

| **Public system** | | | | | |
| --- | --- | --- | --- | --- | --- |
|  | 2024 | 2025 | 2026 | 2027 | 2028 |
| Durvalumab | 15% | 30% | 50% | 65% | 70% |
| SoC (*watch and wait)* | 85% | 70% | 50% | 35% | 30% |
| **Private system** | | | | | |
| Durvalumab | 70% | 80% | 90% | 100% | 100% |
| SoC (*watch and wait)* | 30% | 20% | 10% | 0% | 0% |
