# Supplementary material for: Budget impact analysis of durvalumab consolidation therapy vs no consolidation therapy after chemoradiotherapy in stage III non–small cell lung cancer in the context of the Chilean health care system
Source: PLoS One. 2024 Jul 26;19(7):e0307473. doi: 10.1371/journal.pone.0307473 (PMC11280244; doi:10.1371/journal.pone.0307473)
Supplement: S1 Appendix — (ZIP) [file pone.0307473.s001.zip › S1 Table B.docx]

**S1 Appendix. Table B. Parameters scenario analysis 1**

| **Proportion of patients who receive subsequent treatment** | | |
| --- | --- | --- |
|  | Durvalumab | SoC |
| Pembrolizumab | 8.00% | 8.00% |
| Pemetrexed + Cisplatin | 30.00% | 30.00% |
| Pemetrexed + Carboplatin | 30.00% | 30.00% |
| Alectinib | 8.00% | 8.00% |
| Nivolumab | 8.00% | 8.00% |
| Atezolizumab | 8.00% | 8,00% |
| Cost of subsequent treatment per patient (FONASA) | | |
| Pembrolizumab | $ 77,935 | $ 77,935 |
| Pemetrexed + Cisplatin | $ 815 | $ 815 |
| Pemetrexed + Carboplatin | $ 972 | $ 972 |
| Alectinib | $ 280 | $ 280 |
| Nivolumab | $ 50,597 | $ 50,597 |
| Atezolizumab | $ 33,967 | $ 33,967 |
| Total cost subsequent treatment | $ 13,558 | $ 13,558 |
| Cost of subsequent treatment per patient (ISAPRE) | | |
| Pembrolizumab | $ 155,994 | $ 155,994 |
| Pemetrexed + Cisplatin | $ 3,399 | $ 3,399 |
| Pemetrexed + Carboplatin | $ 3,751 | $ 3,751 |
| Alectinib | $ 464 | $ 464 |
| Nivolumab | $ 74,996 | $ 74,996 |
| Atezolizumab | $ 49,541 | $ 49,541 |
| Total cost subsequent treatment | $ 24,624 | $ 24,624 |
