# Supplementary material for: Budget impact analysis of durvalumab consolidation therapy vs no consolidation therapy after chemoradiotherapy in stage III non–small cell lung cancer in the context of the Chilean health care system
Source: PLoS One. 2024 Jul 26;19(7):e0307473. doi: 10.1371/journal.pone.0307473 (PMC11280244; doi:10.1371/journal.pone.0307473)
Supplement: S1 Appendix — (ZIP) [file pone.0307473.s001.zip › S1 Table C.docx]

**S1 Appendix. Table C. Results scenario analysis (incremental costs) private perspective.**

|  | **2024** | **2025** | **2026** | **2027** | **2028** |
| --- | --- | --- | --- | --- | --- |
| **Scenario 1** | | | | | |
| Drug acquisition cost, USD | 1,413,951 | 2,449,725 | 2,795,132 | 3,145,134 | 3,290,343 |
| Administration cost, USD | 42,495 | 73,624 | 84,005 | 94,524 | 98,888 |
| Adverse event cost, USD | -2,372 | -3,465 | -3,945 | -4,432 | -4,574 |
| Monitoring cost, USD | -70,917 | -212,785 | -291,255 | -317,952 | -298,022 |
| End of life cost, USD | -553 | -1,572 | -2,327 | -2,902 | -3,302 |
| Subsequent treatment cost, USD | -54,453 | -111,169 | -98,188 | -67,947 | -28,636 |
| Total cost | $1,328,150 | $2,194,358 | $2,483,422 | $2,846,425 | $3,054,698 |
| **Scenario 2** | | | | | |
| Total number of patients who have been treated by the end of each year | 31 | 31 | 32 | 32 | 33 |
| Drug acquisition cost, USD | 929,343 | 1,610,122 | 1,837,147 | 2,067,191 | 2,162,632 |
| Administration cost, USD | 27,931 | 48,391 | 55,214 | 62,128 | 64,996 |
| Adverse event cost, USD | -1,559 | -2,277 | -2,593 | -2,913 | -3,007 |
| Monitoring cost, USD | -46,612 | -139,857 | -191,432 | -208,979 | -195,880 |
| End of life cost, USD | -364 | -1,033 | -1,529 | -1,908 | -2,170 |
| Subsequent treatment cost, USD | -22,085 | -41,759 | -36,744 | -24,820 | -10,533 |
| Total cost | $886,654 | $1,473,587 | $1,660,062 | $1,890,699 | $2,016,039 |
| **Scenario 3** | | | | | |
| Drug acquisition cost, USD | 941,902 | 1,631,881 | 1,861,973 | 2,095,126 | 2,191,857 |
| Administration cost, USD | 13,965 | 24,195 | 27,607 | 31,064 | 32,498 |
| Adverse event cost, USD | 7,802 | 11,985 | 11,796 | 11,597 | 11,620 |
| Monitoring cost, USD | 536,848 | 1,590,062 | 2,461,402 | 3,167,298 | 3,167,298 |
| End of life cost, USD | 1,317 | 4,286 | 6,561 | 8,005 | 8,990 |
| Subsequent treatment cost, USD | 57,594 | 130,646 | 107,502 | 87,934 | 79,914 |
| **Budget Impact** | $1,559,428 | $3,393,056 | $4,476,842 | $5,401,025 | $6,092,309 |
